# External Load and Muscle Activation Monitoring of NCAA Division I Basketball Team Using Smart Compression Shorts

**DOI:** 10.3390/s21165348

**Published:** 2021-08-08

**Authors:** David N. Saucier, Samaneh Davarzani, Reuben F. Burch V, Harish Chander, Lesley Strawderman, Charles Freeman, Logan Ogden, Adam Petway, Aaron Duvall, Collin Crane, Anthony Piroli

**Affiliations:** 1 Human Factors & Athlete Engineering, Center for Advanced Vehicular Systems (CAVS), Starkville, MS 39759, USA; burch@ise.msstate.edu; 2 Department of Industrial & Systems Engineering, Mississippi State University, Mississippi State, MS 39762, USA; sd1738@msstate.edu (S.D.); strawderman@ise.msstate.edu (L.S.); 3 Department of Kinesiology, Mississippi State University, Mississippi State, MS 39762, USA; hchander@colled.msstate.edu; 4 Department of Fashion Design & Merchandising, Mississippi State University, Mississippi State, MS 39762, USA; cfreeman@humansci.msstate.edu; 5 Strength & Conditioning, The University of Utah, Salt Lake City, UT 84112, USA; logden@huntsman.utah.edu; 6 Athletic Performance, Washington Wizards, Washington, DC 20004, USA; apetway@monumentalsports.com; 7 Strength & Conditioning, Mississippi State University, Mississippi State, MS 39762, USA; aduvall@athletics.msstate.edu (A.D.); ccrane@athletics.msstate.edu (C.C.); 8 Strength & Conditioning, Tampa Bay Buccaneers, Tampa, FL 33607, USA; apiroli@buccaneers.nfl.com

**Keywords:** court-based sport, surface electromyography, accelerometer, wearable technology, smart garments

## Abstract

There is scarce research into the use of Strive Sense3 smart compression shorts to measure external load with accelerometry and muscle load (i.e., muscle activations) with surface electromyography in basketball. Sixteen external load and muscle load variables were measured from 15 National Collegiate Athletic Association Division I men’s basketball players with 1137 session records. The data were analyzed for player positions of Centers (*n* = 4), Forwards (*n* = 4), and Guards (*n* = 7). Nonparametric bootstrapping was used to find significant differences between training and game sessions. Significant differences were found in all variables except Number of Jumps and all muscle load variables for Guards, and all variables except Muscle Load for Forwards. For Centers, the Average Speed, Average Max Speed, and Total Hamstring, Glute, Left, and Right Muscle variables were significantly different (*p* < 0.05). Principal component analysis was conducted on the external load variables. Most of the variance was explained within two principal components (70.4% in the worst case). Variable loadings of principal components for each position were similar during training but differed during games, especially for the Forward position. Measuring muscle activation provides additional information in which the demands of each playing position can be differentiated during training and competition.

## 1. Introduction

Recently, load monitoring of athletes during training and competition has seen tremendous growth due to the introduction of wearables to the market [1]. Despite this increase, coaches and practitioners have expressed frustration and a lack of trust with some of these technologies due to inconsistent data reporting, poor comfort and fit, and lack of transparency in data calculations [1,2,3]. Load monitoring is reported in terms of external and internal loads using both biomechanical and physiological metrics [4]. External load represents a “dose” component—actions performed by an athlete—while internal load represents a “response” component—the adaptations that come about from the athlete’s actions. External and internal loads have been measured using various methodologies with a lack of consistency in how results are reported [1], in addition to intersystem reliability issues [5], leaving much to be desired when comparing results [1]. Methodologies found in the literature include heart rate monitoring, blood lactate concentration, rating of perceived exertion (RPE) surveys, accelerometers/inertial measurement units (IMUs), global positioning systems, local positioning systems, and optical camera systems.

One gap identified for internal load monitoring for basketball teams is that the biomechanical responses performed during isometric muscle contractions are not being adequately measured [6]. Petway et al. stated that “the physical cost of player-on-player contact loading is a component of basketball that must be examined more thoroughly in future research to more accurately quantify training and competition load” [6]. Biomechanical-based internal load is not commonly measured for load monitoring outside of the use of RPE surveys, which is considered to be a subjective and less direct measure of internal load [4]. To directly and accurately quantify biomechanical internal load, joint contact forces (e.g., the stabilizing muscle forces or muscle–tendon forces) need to be measured, which is currently only feasible in a lab environment [4]. Alternatively, biomechanical internal load could be quantitatively assessed by measuring creatine kinase levels to determine tissue damage, but these are also difficult to measure [4] and implement in a practical setting, as is muscle oxygen saturation (SmO2) testing on a team scale. There is potential that this gap in internal load monitoring could be filled by examining muscle activation using surface electromyography (sEMG). Albeit indirect, the measurement of muscle activation over time could provide insight into the degree to which an athlete is undergoing tissue damage and muscle soreness, providing an additional perspective in which practitioners can assess the state of their athletes.

This paper aims to present the use of a novel, commercially available athlete monitoring system, the Strive Sense3 (Strive), to measure external load and muscle activation during a season of training and competition for a National Collegiate Athletic Association (NCAA) Division I men’s basketball team. Strive is a pair of compression shorts that measures external load using an accelerometer and global positioning system (GPS) placed in a plastic enclosure mounted at the center of the waist band using button snaps, and it measures muscle activation using sEMG electrodes that are embedded into several parts of the fabric. Though conflicting research exists on the validity and reliability of sEMG-embedded compression shorts [7,8], the technology has been observed to have good concurrent validity and good interrater reliability in comparison to a research-grade sEMG system when measuring quadriceps muscle activations as a normalized voltage output during the squat exercise [9]. Other brands of sEMG-based compression shorts have been investigated as well, indicating strong validity and reliability for exercise [10,11,12], activities of daily living [13], and tactical applications (i.e., military and first responders) [14,15]. Several of these studies emphasize that the sEMG electrodes should maintain good contact with the muscle to collect meaningful data. Authors from multiples studies encouraged users of this technology to properly lubricate the areas where the electrodes make contact with the leg muscles for users that do not produce enough sweat and to ensure that the shorts fit the participant well in order to produce a reliable sEMG signal [9,10,16].

The previous literature has found accelerometers measuring accelerations in three directions to have moderate to high test–retest reliability during physical activity and demonstrated convergent validity when compared against oxygen uptake [1,17]. Wearable devices using this technology are commonly worn on the upper thoracic region to improve the GPS signal [1]. However, Strive measures external load using the accelerometer located near the center of mass (COM) directly below the umbilicus. This placement has been suggested as the criterion placement given that it is less susceptible to noise in the vertical vector motion that can be introduced from upper body movements such as shoulder-girdle sway, arm swing, and trunk flexion [1,17,18].

Additionally, this technology has yet to be presented in the literature for load monitoring during an entire season of training and competition for a basketball team [1,6]. Since this type of wearable technology is just beginning to be used in the field for high-level athletes, there is little to no research into the effectiveness of sEMG technology for detecting trends and providing metrics from which coaches can make decisions to modify training regimens and competition strategies based on objective, measured data. Thus, an analysis of the data collected from Strive will be conducted and compared to other load monitoring studies in the field. Key relationships that will be investigated include a comparison of sEMG and accelerometer measurements between training and game sessions, as well as differences in variable loadings between player positions. Finally, sEMG and accelerometer measures are discussed from a practitioner perspective, addressing how the data collected from Strive can be used to adjust the periodization and intensity of training sessions prior to games, and to assess differences in player position demands between training and game sessions.

## 2. Materials and Methods

### 2.1. Participants

Data collected from 15 NCAA Division I male basketball players (age 21.20 [1.80] y, height 201.75 [8.33] cm, body mass 97.85 [10.52] kg) were examined for this study. These data were recorded from players during the regular season for both practice (training sessions) and competition (game sessions). Data were not recorded from players for a specific session if they could not fully participate in a session (i.e., injury) or participated for less than 5 min in a session. All participant data were anonymized prior to analysis. This exempt study was approved by the university’s institutional review board (Protocol ID: IRB-21-294).

### 2.2. Design

This research was conducted using an observational study design in which the Strive system collected data from participants during training and game sessions during the 2019–2020 season [19,20,21,22]. Both muscle activation (sEMG) and external load (accelerometer) were measured by the shorts when monitoring player activity for each session. The season lasted for 23 weeks and included 77 practice sessions and 35 competitive game sessions. The data observed for analysis did not include weightlifting sessions performed by the athletes, which would last for 30–60 min. Regular training, or “practice sessions”, would last for 60–180 min, and game session times would vary widely for individual athletes. Practice sessions were designed based on minutes played, player status (starter vs. bench player), position, injury/fatigue status, and difficulty of opponent in the next competitive game. Initially, 932 samples were collected from training sessions and 282 samples from game sessions. When outlier samples were observed, the strength coach would consult with Strive to discuss which results were expected and which were abnormal to ensure that future training decisions were made based on properly collected data. After preprocessing of the data to remove outliers, samples were reduced by 6.1% from 932 to 875 samples for training sessions and by 7.1% from 282 to 262 samples for game sessions after removing outliers, resulting in a total of 1137 samples. Players were grouped into the positions of Guards (*n* = 7; 573 samples), Forwards (*n* = 4; 295 samples), and Centers (*n* = 4; 269 samples).

### 2.3. Procedures

The basketball players’ shorts that would normally be used during the season were outfitted by Strive with the sEMG pads and button snaps on the waistband to attach the enclosure containing the electronic components. Upon initially donning the retrofitted shorts and receiving feedback from the athletes regarding comfort, short sizes were adjusted as requested by the athletes. The sEMG pads embedded in the shorts are placed in the muscle group areas of the rectus femoris, biceps femoris, and gluteus maximus to measure muscle activations in the left and right legs. Data collected from the shorts were recorded on an Apple iPad for each session using Strive’s propriety iPhone OS (iOS) application, Strive Performance.

During training and game sessions, data were included from warm-ups and rest periods such as timeouts and substitutions. Between measurements collected from both the accelerometer and sEMG pads, 74 variables were computed for each session. These variables were calculated based on measures collected from the accelerometer at 100 Hz and sEMG pads at 1024 Hz. The authors narrowed the analysis down to seven external load and five muscle load variables based on what was found in the literature as well as what was of interest to the coaching staff: Total Distance [6,23], Average Speed [6,24], Average Max Speed [6,25], Number of Jumps [1,6,26], Number of Accelerations [1,6,26], Number of Decelerations [1,6,26], Number of High Accelerations [1,6,26] (accelerations measured at greater than 3.5 m/s^2^), Number of High Decelerations [1,6,26] (decelerations measured at greater than 3.5 m/s^2^), Total Quad (Quadriceps) Muscle, Total Hamstring Muscle, Total Glute (Gluteus) Muscle, Total Left Muscle, and Total Right Muscle. Table 1 contains a description of how these variables are computed. Three additional summary variables reported by Strive—Muscle Load (sum of muscular activation from all sEMG sensors, divided by a scaling factor), Traditional Player Load (sum of accelerations across all axes of the accelerometer during movement, divided by a scaling factor), and Efficiency (ratio of Traditional Player Load to Muscle Load)—were analyzed.

### 2.4. Statistical Analysis

Python 3 was used to preprocess and analyze the data collected from Strive. In the preprocessing phase, any samples that had an active time of 0 ms were removed. Additionally, samples where Muscle Load and Efficiency values exceeded two standard deviations from the mean were removed. This was due to outliers that were observed and resulted from either sEMG pads not making good contact with the muscle group (abnormally low output) or from general wear and tear of the sEMG sensor pad through repeated use (abnormally high output).

Most variables did not follow a normal distribution and/or did not have equal variance for the measurements in training and game sessions, which are identified in Table 2. The Shapiro–Wilk test was used to check the normality assumption [27], and Levene’s test was used to assess the equality of variances between training and practice sessions [28]. Therefore, a nonparametric bootstrapping method [29] was used instead. This approach is based on resampling and does not hold the normality distribution and equality of variance assumptions that are required for other statistical methods. Two-sample bootstrap hypothesis testing was carried out on Traditional Player Load, Muscle Load, Efficiency, eight external load variables, and five muscle load variables to evaluate the equality of the mean of each variable between training and game sessions. The 95% confidence intervals (CI) based on the bootstrap percentiles while resampling 10,000 times are provided for the difference in each variable between training and game sessions.

Principal component analysis, or PCA, was used to extract the principal components (PCs) that account for the variation in external load. PCA results can indicate which variables contribute the most to the variation in external load for each playing position [26]. While original variables could be correlated with each other, PCs are independent and orthogonal to each other, and they correspond to directions of the maximum variation in the original observations [30]. PCA transforms the original data into a new space in which the dimension of the dataset could be reduced by preserving the first few PCs and eliminating the rest without losing much information.

Loadings (in terms of statistical analysis, not dose–response monitoring of players) are considered as the coefficients of regression between the original variables (independent variables) and the corresponding PC (dependent variable). The loading of external load variables on each PC was evaluated to determine how the PCs are correlated with these variables. A varimax rotation was applied to the loadings to make correlations between rotated PCs and original variables more distinct and easier to interpret [26]. Data were normalized within-subject before PCA. Kaiser–Meyer–Olkin (KMO) and Bartlett’s sphericity [31] tests were performed to determine the sampling adequacy and usefulness of PCA for these data. KMO values greater than 0.5 indicate that the data are eligible for PCA, which was the case for all positions in training and games. All values of the Bartlett test were significant, indicating PCA could be useful with these data. Only PCs with eigenvalues greater than 1 were used [26].

## 3. Results

Bootstrapping results for testing the equality of means over training and game sessions (*p* < 0.05) are presented in Table 3. The 95% CI represents an estimate of the difference in means between game and practice sessions, estimated within a lower and upper bound. It shows that with 95% confidence, the difference between the mean value of each variable in the training and game would be within the specified range. *p*-Values less than 0.05 indicate that there is a statistically significant difference between the means of training and game sessions. The effect size was computed as Hedges’ g due to the datasets not meeting the homogeneity of variance assumption and also having different sample sizes between training and game sessions [32,33]. Since this study was conducted with highly trained individuals, the following guideline was used for interpretation of effect sizes: trivial (ES < 0.25), small (>0.25 and <0.5), moderate (>0.5 and <1.0), and large (>1.0) [34]. For the Centers, the differences between mean values of Average Speed, Average Max Speed, Total Hamstring Muscle, Total Glute, Total Left Muscle, and Total Right Muscle were significant over training and game sessions, and the rest of the variables had equal means for these two session types. Evaluating the results for the Forwards indicated that only Muscle Load did not have equal means over training and game sessions. Results for the Guard position differed in that all variables except the Number of Jumps and the Total Muscle variables were significantly different between the training and game sessions.

**Figure 1:**
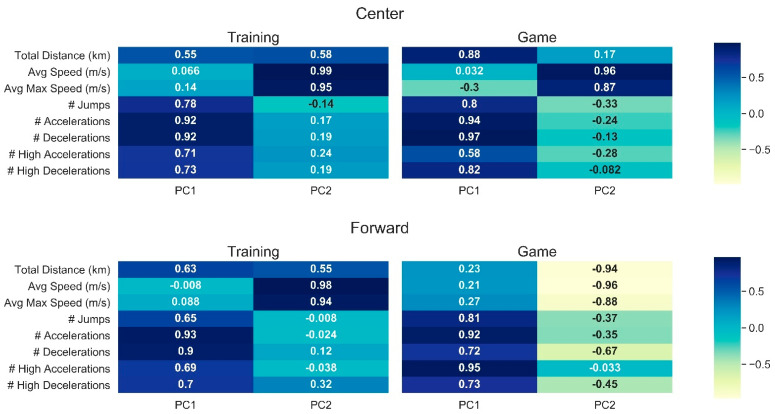

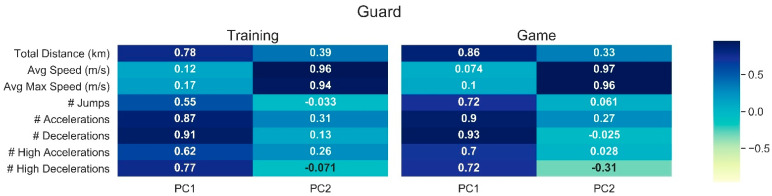
Heatmaps of original variable loadings on each PC for Center, Forward, and Guard playing positions. **Left panels** indicate the results for practice sessions, and **right panels** correspond to the game sessions.

The eigenvalues, explained variance, and cumulative explained variance computed for PCA are presented in Table 4. Two components were retained for each model, which account for more than 70% of the variance in the data. The heatmaps in Figure 1 illustrate the loadings of variables in each PC after applying varimax rotation. Values greater than 0.7 are considered an indicator of a high loading of a variable in a PC [26]. Similar loading patterns were observed for each playing position during training, while distinct patterns were observed during game sessions.

## 4. Discussion

When observing the bootstrapping results, Muscle Load was higher in training for both Forwards and Guards, but not for Centers. This may be a component of technical demands (e.g., cutting and change in direction on the perimeter) or tactical situations (e.g., less ball screen situations decreasing the opportunity to accumulate Muscle Load for Centers). However, for this dataset, the NCAA men’s basketball team used in this study had a high number of “bigs”, or Centers (*n* = 4), meaning their total repetitions during training were generally lower than any given Guard.

Many studies have investigated load data using statistical hypothesis testing methods. In these studies, researchers investigated whether there is a statistically significant difference for load variables during training and game sessions or over various positions. Reina et al. [19] performed load monitoring for a women’s amateur basketball team and found a significantly higher load during games for all external and internal load variables except for the number of impacts per minute. Fox et al. [35] compared external and internal load data from semi-professional male basketball players during physical conditioning training (PCT), games-based training (GBT), and games. They found that all external load variables and summated heart rate zones were significantly higher in PCT and GBT than in games, while the ratio of session rating of perceived exertion (sRPE)/PlayerLoad (external) was the opposite. Heishman et al. found no significant positional difference in external load variables during preseason training [21]. However, this could have been a result of the training style, where “stretch four” players were trained as hybrid positions [21], and these differences may not reveal themselves until in-game performance. Additionally, Ransdell et al. found that PlayerLoad per minute was higher in Guards compared to Posts (Center/Forward hybrid) for multiple years of data collection [22].

When observing the bootstrapping results, the results of this study indicate that several load variables were higher during game sessions only for certain positions. When compared to training sessions, game sessions resulted in higher Muscle Load, Traditional Player Load, and all Total Muscle variables for Centers. Traditional Player Load, Efficiency, six external load variables, and all Total Muscle variables were higher in game sessions for Forwards. All external load variables except Number of Jumps, Efficiency, and Traditional Player Load were higher or equivalent in games for Guards. Additionally, only the Total Glute and Total Right Muscles were higher for Guards during games by a small margin.

During competition, Guards have previously been found to run longer distances than Centers and Forwards [25], which agrees with the results found in this study (Centers: 1.67 ± 0.83 km; Forwards: 3.86 ± 1.95 km; Guards: 4.18 ± 1.39 km). In contrast to the results of Svilar et al., which found that Centers recorded a higher amount of accelerations than Forwards and Guards during training [26], the results of this study indicate that these measures were lower for Centers in comparison to Guards and Forwards (Centers: 112.75 ± 51.93; Forwards: 151.16 ± 56.25; Guards: 164.96 ± 59.88). This could be a result of the different training plans used between these studies.

As Petway et al. pointed out, while accelerometry data can be useful, the technology becomes limited when trying to measure the metabolic demands of isometric muscle contractions that occur during player-on-player contact, since there is little movement taking place for the accelerometer to detect [6]. Given that this is the first study of this nature to observe muscle activation data over the course of a season of basketball, there is not much precedent in terms of comparing results to other studies for muscle activation data specifically. The heart rate, a measure of internal load, could be compared to muscle activation. Gocentas et al. found that the maximum heart rate, on average, was slightly higher for Guards than Forwards during training [36]. This agrees with the results found in this study, where Muscle Load for Guards was higher (450.34 ± 221.31) than that for Forwards (381.28 ± 173.82) in training. There is potential that these two metrics could be correlated. However, further research is warranted where both heart rate and muscle activation monitoring devices are worn concurrently to conduct a valid comparison.

Muscle Load was higher in training for both Forwards and Guards, but not for Centers. This may be a component of technical demands (e.g., cutting and change in direction on the perimeter) or tactical situations (e.g., less ball screen situations decreasing the opportunity to accumulate Muscle Load for Centers). However, for this dataset, the NCAA men’s basketball team participating in this study had a high number of “bigs”, or Centers (*n* = 4), meaning their total repetitions during training were generally lower than any Guard or Forward as there are generally two Guard and Forward positions practicing or playing at any time vs. one Center. For specific muscle group variables, Centers and Forwards had higher Total Muscle measurements in games for all variables. However, Total Muscle variables for Guards were either roughly the same or lower during games when compared to training.

The results for individual muscle groups indicate that Centers tend to favor using their quadriceps over the other muscle groups, whereas Guards and Forwards tend to favor the hamstrings. During competition, this differs in that Centers and Forwards become hamstring-dominant, whereas Guards tend to favor both the hamstrings and the glutes. Observing the results of the total muscle activity measurements collected from the left and right legs can provide an insight into the athletes’ muscle asymmetry. This reveals that muscle asymmetry was more present, on average, during training for both Centers (46.0% L/54.0% R) and Forwards (46.4% L/53.6% R) when compared to Guards (48.6%L/51.4%R). During competition, this difference in asymmetry increases for all positions, with Centers (45.5% L/54.5% R) experiencing the smallest change, Forwards experiencing the largest change (44.2% L/55.8% R), and asymmetry for Guards changing moderately (47.0% L/53.0% R). This increase in asymmetry for all positions could indicate that the demands of competition are pushing the athlete to rely more on their dominant leg. Regarding leg dominance by position, the side of the court most played upon by the individual players may impact this measurement, as well as other actions such as shielding a defender or backing someone down to gain advantage in the post. Guards had the lowest amount of right leg dominance, and they also play in the position that controls the game speed, often playing from both sides of the court.

When observing the effect size, there are several variables to note regarding differences between positions. For Muscle Load, a trivial effect size was observed for Forwards, while a small effect size was observed for Guards and Centers. The effect size was due to an increase in Muscle Load for Centers and a decrease for Guards when comparing training to competition. If the goal is for the strength and conditioning staff to match the demands of competition during training, then this indicates Guards were being overtrained and Centers were being undertrained. For this team, the Guards were intentionally trained to experience greater Muscle Load in practice since it was expected that there would be fewer Guards overall that would be playing during competition. This consequently led to a greater demand for Guards during practice since the position requires them to be in the best shape and most efficient with their movement during competition. A large effect size was observed for Traditional Player Load for both Guards and Forwards in comparison to the trivial effect size of the Centers. This is likely due to the high acceleration and high speed demands prompted by these positions. Since the Centers for this team played a traditional role rather than a hybrid position, they largely focused on playing close to the basket and are characterized as being the largest, slowest players. The high-velocity and high-intensity movement required for the Guard position is reflected in the moderate effect sizes found in the number of Accelerations, High Accelerations, and High Decelerations. This shift from training to competition was larger in comparison to the demands imposed for the Center and Forward positions.

In regard to the PCA heatmaps in Figure 1, the interpositional variances are reasonable when examining both the technical and tactical demands of training and games [20,26,37,38]. External load among Centers is dictated by court transitions and ball screen situations (i.e., accelerations and decelerations). Additionally, the number of jumps under the goal as well as speed to quickly move to both defensive and offensive positions “in the paint” are other noted physical parameters for Centers. All these physical parameters are noted in Figure 1 as carrying the most weight in explaining the variance for external load and are commonly weighted for Centers between practice and games. Guards must be quick and agile; therefore, the impetus for external load is speed and jumping ability. Consequently, the same weighting as well as commonalities between practice and games for Guards is visualized in Figure 1. Finally, Forwards play in a hybrid position and must possess a broad array of physical parameters (i.e., endurance and speed) for in-game performance, and their external load during games may vary based on the opponent and specific demands of each individual competition which could explain how practice and game demands differed greatly for the second component weight in Figure 1. The similarity in the loading of the training sessions could be explained by the training activities, which might be less technical and generally train all the players in various positions. Previous research has also investigated the differences in training and match play [35,39].

Svilar et al. [26] performed PCA on the total and high-intensity external load variables including jumping, acceleration, deceleration, and change in direction for various playing positions in training sessions. They suggested that two to three PCs could explain the external training load for each playing position, which could be used to help coaches enhance their training programs. In our analysis, all positions had similar loadings during training sessions, but different loading configurations during games. Further, two PCs explained anywhere from 70 to 87% of the variance in the data, whereas the PCA performed by Svilar et al. explained 100% of the variance in the data within three PCs for Guards and Forwards and 96% of the variance within three PCs for Centers [26]. This could be due to the differences in the positioning of the accelerometer as well as the algorithms used to compute these variables in different devices [6,26].

### 4.1. Application for Coach Practitioners

The last five authors of this study are coaches at the collegiate and professional levels of athletics and will use an autoethnographic frame to speak as practitioners for the research findings, Strive Sense3 technology, and implications for monitoring, training, and recovery of athletes [9,40]. Using a technology such as Strive, the specific game demands required of athletes during competition can be captured. The data identifying these demands can then be used to build conditioning session progressions which can then be tracked and assessed to ensure practice sessions consider the necessity of both the preparation intensity and rest and recovery periods. A baseline of seasonal data not only allows coaches to assess the potential risk of de-training in-season but it also provides greater measures of what true “return to play” is for injured athletes. Seasonal baseline data enable strength coaches and trainers to formulate an autoregulated plan for athletes to go through the “return to stages of recovery” [41]. With an accurate understanding of the demands required for an entire season of competition with data that house more than just the commonly captured external load—such as including the muscle activation component—true game demands can be progressively built upon throughout the preseason. Proper acute-to-chronic workload ratios can be designed from the experience of the strength coach practitioner and then compared to the demands of the previous season while taking each individual athlete’s progression history into account. For example, if a coaching practitioner knows certain drills may give the team an average muscle activation value (i.e., Muscle Load = 300) representative of game demands, the coach can use certain drills or pieces of sessions to progressively build the athletes up to game demands.

While, generally, injuries cannot be prevented, they can be mitigated, and a tool such as Strive enables coach practitioners to investigate whether an athlete is at risk of injury. Utilizing specific data trends such as looking at 3-, 7-, and 14-day timelines can be used to investigate the beginnings of indicators of injuries such as stress fractures, soft tissue injuries, and countless corrections of poor recovery habits. Coaches can proactively look for overuse and underuse of specific muscle groups. Through the collection of muscle activation data, a deeper dive may sometimes uncover a more serious issue that the athlete may have but not report as it would hold them out of training and games, thereby leading to longer-term injury recovery.

The high standard deviation for muscle activation and loads in this study suggests the coaching staff did a good job at undulating loads of practices. Both the strain and monotony of training with the reported load values should be within a reasonable limit which can only be realistically determined by the coaching staffs’ contextual understandings regarding the play style of the team and the individual athlete regimen needs. Therefore, findings regarding the differences between practice and game demands as well as between the different skill positions could be helpful to coaches and practitioners regarding sequencing of training menu items to reflect match play, as well as creating an optimal environment for in-game performance during practices.

Additional strategies can be employed in a more fixed environment, such as weight training sessions. Traditionally, weight training sessions are measured in volume (e.g., the number of workout sets times the number of weightlifting repetitions) and intensity (i.e., percentage weight from the athlete’s one repitition maximum). The Strive Sense3 can be used to provide an additional layer of measuring the overall demands of a weight training session. The key to any training program is having sound principles of periodization and monitoring the fluctuation in various stressors. Outside of laboratory gold standards, there are currently not many reliable and rugged tools for measuring muscle load during a training session.

In practice, the strength coach that utilized the Strive shorts for this study used the data to monitor the return to play status for specific athletes using the Traditional Player Load, Muscle Load, and Distance variables. They prescribed specific periodization parameters and goals for the athletes based on the position group averages for each training session. The athletes needed to reach benchmarks set by the strength coach and certified athletic trainer (ATC) without feeling pain so that they could recover properly before moving to the next benchmark. In one example, a lower limb injury was detected early on when looking at the right-to-left leg muscle symmetry and muscle load variables. It was noted that the symmetry was shifting from one leg to another, and muscle load was increasing when observing 7-day and 14-day trends, which was not normal for the athlete being assessed. The athlete would normally perform with even symmetry (50% R/50% L). After hearing from the athlete that they were dealing with foot pain following practice and games, a shift was found towards the left leg in the next practice (52% L/48% R). The strength coach noticed a trend as he monitored the athlete for one more practice, which resulted in extreme asymmetry (64% L/36% R), and therefore the athlete was removed from practice for the week. The athlete was still included for the next game several days later but was again performing with an abnormal asymmetry (58% L/42% R) and was removed after the start of the second half. He was then evaluated by the ATC, who found the start of a lower limb injury. The athlete began rehabilitation under guidance from the ATC in combination with the return to play protocols used with the Strive shorts. This included performing rehabilitation exercises that would match the Muscle Load demands normally experienced in practice but replaced with isometric contraction exercises such as wall sits and squat holds. The player was able to return in three weeks without any further issues.

### 4.2. Limitations

Proper fitting and wetting of sensors are important components when utilizing Strive [9]. Of necessary consideration when evaluating wearable sensor adoption is fit and perceived comfort. For athlete adoption of wearable sensors, prevention of discomfort at sensor locations will increase overall user satisfaction. Prior studies indicated the importance of the fabric, fit, and comfort factors when investigating the validity of wearable sensor data [42,43,44]. As noted in the methods, athletes’ shorts were fitted based on personal preference and adjusted when the shorts felt too tight after embedding the sEMG pads. If short sizes were increased too much, this could have led to a looser fit and consequently result in less accurate sEMG recording where the output was lower than normal. Therefore, this tradeoff between comfort and optimal sensor placement must be considered.

Further, when an abnormally high output was observed due to wear and tear of the sensor pads, the shorts would also have to be replaced. Outlier removal was conducted to mitigate potential error from these issues, but proper education and consulting of the technology from the vendor should be carried out for the coaching staff to better understand when to identify abnormal data recordings. The validity and reliability of sEMG outside of a laboratory setting have been largely unexplored. It is unknown how much player-on-player contact, individual anthropometry, and textile selection can influence the recorded output. Lastly, acceleration and deceleration values were higher for some positions than what has typically been found in previous studies during match play [6]. A comparison to values found in the previous literature is shown in Table 5. This difference could be due to the placement of the accelerometer when compared to other technologies which have been placed on the upper thoracic region, at the hip, or on a chest strap [1].

### 4.3. Future Work

Future research into the comfort and fit of Strive is warranted to ensure that a proper fit can be achieved while still collecting valid and reliable data. Additionally, the analysis in this study utilized a small portion of the total variables recorded by Strive. A deeper exploratory analysis utilizing advanced statistical and machine learning techniques could reveal other patterns that are difficult to observe when only considering several variables. For future studies, qualitative data such as rating of perceived exertion surveys could be examined alongside the data collected from the quantitative wearables data and be analyzed using a mixed methods approach in combination with temporal pattern (T-pattern) analysis [45,46]. Additional hypothesis testing could be conducted to directly compare player positions and individuals to each other, and data visualization techniques such as k-means clustering [47], histograms [26], and radar charts [48,49,50] could be used to distinguish how individual players compare in the variables measured. Relationships should be investigated between muscle activation and methods commonly used to measure internal load such as heart rate monitoring and sRPE surveying. Data trends over time and how they are affected by periodization and adjustments in training regimens should be investigated at a more granular level (e.g., week-by-week analysis and individual player profiles). Future research that would be of interest to strength and conditioning practitioners in this field should investigate what training methods and modalities can positively influence optimal lower body muscle recruitment ratios such as evaluating leg asymmetry and comparing quadriceps activations to glute activations.

## 5. Conclusions

When comparing measures taken between training and games, Centers were found to have significantly different speeds and muscle activations, Forwards were found to have significant differences across all measures except Muscle Load, and Guards were found to have significant differences for Muscle Load and all external load variables except Number of Jumps. These findings suggest that each position in basketball has its own unique external load and muscle activity profile, and that training regimens can be adapted to match these demands in competition. Observing the PCA results indicates that the external load profiles of positions were similar in training but differed considerably for Forwards in comparison to Centers and Guards. This analysis provides insight into which variables account for the most variance in player positions, suggesting that acceleration and deceleration count variables tend to be more distinguishable of player profiles rather than average speed measures for all player positions and jump count for Forwards.

Collecting various external load measures at the waist while also measuring muscle activation of the legs during training and games of basketball can provide a clearer picture of the state of the athlete. Consequently, training can be adjusted for each playing position’s match play demands based on what was observed during games, rather than keeping demands similar for all positions during training. The analysis of muscle activation provides an additional layer of information in which playing positions can be differentiated during games, giving coach practitioners another perspective that can be used to better optimize training for their athletes. This novel wearable has potential to bring a new layer of baselining performance that can be used by coach practitioners to better prepare, train, and rehabilitate their athletes.

## Figures and Tables

**Table 1:** List of descriptions for each of the load variables used for analysis.

Variable	Computation
Total Distance	Estimated based on cadence and stride length. Cadence is defined as the step count detected by the accelerometer.
Average Speed	Speed over each second is averaged to a single value (reducing sampling to 1 Hz), and the average of these values is reported.
Average Max Speed	Top speed over a second is averaged to a single value (reducing sampling to 1 Hz), and the average of these values is reported.
Number of Jumps	Jump is counted as any jump measured greater than 175 mm off the ground.
Number of Accelerations	Total amount of accelerations measured greater than or equal to 1.42 m/s^2^.
Number of Decelerations	Total amount of decelerations measured greater than or equal to 1.42 m/s^2^.
Number of High Accelerations	Total amount of accelerations measured greater than or equal to 3.5 m/s^2^.
Number of High Decelerations	Total amount of decelerations measured greater than or equal to 3.5 m/s^2^.
Total Quad Muscle	Sum of sEMG values recorded over time for the left and right quadriceps muscle sensors.
Total Hamstring Muscle	Sum of sEMG values recorded over time for the left and right hamstring muscle sensors.
Total Glute Muscle	Sum of sEMG values recorded over time for the left and right glute muscle sensors.
Total Left Muscle	Sum of sEMG values recorded over time for the quadriceps, hamstring, and glute muscles in the left leg.
Total Right Muscle	Sum of sEMG values recorded over time for the quadriceps, hamstring, and glute muscles in the right leg.

**Table 2:** Table of variables not meeting assumptions required for analysis of variance (ANOVA).

Variable	Training Sessions Follow Normal Distribution	Game Sessions Follow Normal Distribution	Session Types Have Equal Variance
Traditional Player Load	Yes	No	No
Total Distance	No	No	No
Average Speed	No	No	No
Average Max Speed	No	No	No
Number of Jumps	No	Yes	No
Number of Accelerations	No	No	No
Number of High Accelerations	No	No	No
Number of High Decelerations	No	No	No
Muscle Load	No	No	No
Efficiency	No	No	No
Total Quad Muscle	No	No	Yes
Total Hamstring Muscle	No	No	No
Total Glute Muscle	No	No	Yes
Total Left Muscle	No	No	Yes
Total Right Muscle	No	No	No

**Table 3:** Bootstrapping results for equality of means between training and game sessions. The variables that are statistically significant (*p* < 0.05) are in bold. Effect size calculated using Hedges’ g. Guideline for interpreting effect size: trivial (ES < 0.25), small (>0.25 and <0.5), moderate (>0.5 and <1.0), and large (>1.0) [34].

Player Position	Load Factor (Unit)	M_training_ (SD_training_)	M_game_ (SD_game_)	*p*-Value	95% CI (M_game_–M_training_)	Effect Size (M_game_–M_training_)
Centers	Muscle Load	386.64 (197.81)	437.20 (220.76)	0.079	[−7.69, 109.05]	0.25
	Traditional Player Load	370.09 (118.79)	399.10 (182.99)	0.137	[−16.25, 75.73]	0.21
	Efficiency	1.23 (0.79)	1.09 (0.66)	0.180	[−0.34, 0.06]	0.18
	External load factors:					
	Total Distance (km)	1.72 (0.84)	1.67 (0.83)	0.692	[−0.28, 0.18]	0.06
	**Average Speed (m/s)**	**2.02 (0.40)**	**1.88 (0.31)**	**0.012**	**[−0.23, −0.05]**	0.37
	**Average Max Speed (m/s)**	**3.29 (0.31)**	**3.14 (0.25)**	**<0.001**	**[−0.22, −0.07]**	0.51
	Number of Jumps	59.14 (29.05)	53.71 (23.12)	0.168	[−12.17, 1.42]	0.20
	Number of Accelerations	112.75 (51.93)	98.42 (65.30)	0.069	[−31.15, 2.41]	0.26
	Number of Decelerations	122.54 (60.65)	106.41 (67.37)	0.072	[−34.18, 2.18]	0.26
	Number of High Accelerations	7.06 (5.35)	5.76 (5.35)	0.090	[−2.77, 0.20]	0.24
	Number of High Decelerations	7.84 (7.56)	6.89 (6.56)	0.368	[−2.84, 0.98]	0.13
	Muscle load factors:					
	Total Quad Muscle	1.86 × 10^5^ (1.57 × 10^5^)	2.09 × 10^5^ (1.47 × 10^5^)	0.294	[−1.84 × 10^4^, 6.51 × 10^4^]	0.15
	**Total Hamstring Muscle**	**1.73 × 10^5^ (1.10 × 10^5^)**	**2.80 × 10^5^ (2.12 × 10^5^)**	**<0.001**	**[5.57 × 10^4^, 1.62 × 10^5^]**	0.75
	**Total Glute Muscle**	**1.70 × 10^5^ (9.83 × 10^5^)**	**2.34 × 10^5^ (1.54 × 10^5^)**	**<0.001**	**[2.55 × 10^4^, 1.05 × 10^5^]**	0.53
	**Total Left Muscle**	**2.44 × 10^5^ (1.49 × 10^5^)**	**3.29 × 10^5^ (2.13 × 10^5^)**	**<0.001**	**[3.20 × 10^4^, 1.42 × 10^5^]**	0.51
	**Total Right Muscle**	**2.86 × 10^5^ (1.86 × 10^5^)**	**3.94 × 10^5^ (2.52 × 10^5^)**	**<0.001**	**[4.46 × 10^4^, 1.75 × 10^5^]**	0.30
Forwards	Muscle Load	381.28 (173.82)	378.80 (191.27)	0.920	[−51.35, 50.56]	0.01
	**Traditional Player Load**	**494.48 (135.39)**	**642.72 (244.11)**	**<0.001**	**[86.08, 208.62]**	0.89
	**Efficiency**	**1.55 (0.76)**	**1.99 (1.03)**	**<0.001**	**[0.18, 0.70]**	0.53
	External load factors:					
	**Total Distance (km)**	**3.02 (1.17)**	**3.86 (1.95)**	**<0.001**	**[0.35, 1.33]**	0.61
	**Average Speed (m/s)**	**2.19 (0.21)**	**2.09 (0.22)**	**0.001**	**[−0.16, −0.04]**	0.47
	**Average Max Speed (m/s)**	**3.31 (0.18)**	**3.18 (0.18)**	**<0.001**	**[−0.17, −0.08]**	0.72
	**Number of Jumps**	**56.65 (31.05)**	**70.18 (28.04)**	**0.002**	**[5.7, 21.3]**	0.44
	**Number of Accelerations**	**151.16 (56.25)**	**177.64 (117.41)**	**0.011**	**[−1.83, 55.79]**	0.36
	**Number of Decelerations**	**153.14 (56.80)**	**182.58 (91.42)**	**0.002**	**[6.59, 52.65]**	0.45
	**Number of High Accelerations**	**14.55 (10.08)**	**19.06 (19.87)**	**0.012**	**[−0.31, 9.64]**	0.35
	**Number of High Decelerations**	**8.74 (6.53)**	**11.03 (8.05)**	**0.017**	**[0.17, 4.37]**	0.33
	Muscle load factors:					
	**Total Quad Muscle**	**1.83 × 10^5^ (1.17 × 10^5^)**	**2.19 × 10^5^ (1.44 × 10^5^)**	**0.037**	**[−1.23 × 10^3^, 7.47 × 10^4^]**	0.29
	**Total Hamstring Muscle**	**1.96 × 10^5^ (1.16 × 10^5^)**	**2.34 × 10^5^ (1.72 × 10^5^)**	**0.036**	**[−3.68 × 10^3^, 8.28 × 10^4^]**	0.29
	**Total Glute Muscle**	**1.63 × 10^5^ (8.39 × 10^5^)**	**2.11 × 10^5^ (1.21 × 10^5^)**	**<0.001**	**[1.85 × 10^4^, 8.04 × 10^4^]**	0.51
	**Total Left Muscle**	**2.51 × 10^5^ (1.39 × 10^5^)**	**2.93 × 10^5^ (1.84 × 10^5^)**	**0.049**	**[−5.06e + 03, 9.12 × 10^4^]**	0.28
	**Total Right Muscle**	**2.90 × 10^5^ (1.66 × 10^5^)**	**3.70 × 10^5^ (2.42 × 10^5^)**	**0.002**	**[1.90 × 10^4^, 1.45 × 10^5^]**	0.43
Guards	**Muscle Load**	**450.34 (221.31)**	**382.86 (178.09)**	**0.001**	**[−103.52, −30.23]**	0.32
	**Traditional Player Load**	**478.39 (126.43)**	**646.22 (176.09)**	**<0.001**	**[134.74, 199.65]**	1.21
	**Efficiency**	**1.35 (0.75)**	**1.99 (0.96)**	**<0.001**	**[0.47, 0.82]**	0.80
	External load factors:					
	**Total Distance (km)**	**3.13 (0.95)**	**4.18 (1.39)**	**<0.001**	**[0.79, 1.30]**	0.99
	**Average Speed (m/s)**	**2.39 (0.22)**	**2.50 (0.15)**	**<0.001**	**[0.07, 0.14]**	0.53
	**Average Max Speed (m/s)**	**3.54 (0.18)**	**3.61 (0.15)**	**<0.001**	**[0.04, 0.10]**	0.40
	Number of Jumps	69.98 (40.28)	69.62 (20.24)	0.943	[−5.39, 4.78]	0.01
	**Number of Accelerations**	**164.96 (59.88)**	**206.08 (75.97)**	**<0.001**	**[27.1, 55.47]**	0.64
	**Number of Decelerations**	**153.56 (55.48)**	**179.65 (71.57)**	**<0.001**	**[12.82, 39.35]**	0.44
	**Number of High Accelerations**	**13.74 (10.05)**	**22.50 (12.41)**	**<0.001**	**[6.46, 11.12]**	0.82
	**Number of High Decelerations**	**11.84 (8.44)**	**17.78 (11.97)**	**<0.001**	**[3.77, 8.21]**	0.63
	Muscle load factors:					
	Total Quad Muscle	1.90 × 10^5^ (1.16 × 10^5^)	1.86 × 10^5^ (1.10 × 10^5^)	0.692	[−2.59 × 10^4^, 1.73 × 10^4^]	0.04
	Total Hamstring Muscle	2.25 × 10^5^ (1.28 × 10^5^)	2.16 × 10^5^ (1.14 × 10^5^)	0.456	[−3.16 × 10^4^, 1.47 × 10^4^]	0.07
	Total Glute Muscle	2.11 × 10^5^ (1.15 × 10^5^)	2.21 × 10^5^ (1.02 × 10^5^)	0.340	[−9.24 × 10^3^, 3.18 × 10^4^]	0.06
	Total Left Muscle	3.04 × 10^5^ (1.71 × 10^5^)	2.93 × 10^5^ (1.53 × 10^5^)	0.480	[−4.16 × 10^4^, 1.97 × 10^4^]	0.07
	Total Right Muscle	3.22 × 10^5^ (1.79 × 10^5^)	3.31 × 10^5^ (1.63 × 10^5^)	0.636	[−2.34 × 10^4^, 4.09 × 10^4^]	0.05

**Table 4:** Eigenvalues and explained variance of preserved principle components (PCs) (eigenvalue greater than one) for external training load factors in the basketball team for each playing position in the training and game sessions.

Playing Position	Training			Game		
	Eigenvalue	% of Variance Explained	Cumulative %	Eigenvalue	% of Variance Explained	Cumulative %
Center	4.26	52.97	52.97	4.66	57.37	57.37
	1.81	22.55	75.52	1.65	20.30	77.66
Forward	3.77	46.95	46.95	5.68	69.95	69.95
	1.94	24.13	71.08	1.44	17.75	87.71
Guard	4.08	50.83	50.83	4.08	50.61	50.61
	1.57	19.59	70.42	2.01	24.98	75.58

**Table 5:** Comparison of acceleration and deceleration count variables measured during game sessions from presented dataset to other datasets found in the literature [6]. Measurements under playing positions in which the mean was higher than the maximum value of typical results are in bold.

Variable Measured	Centers (Mean ± SD)	Forwards (Mean ± SD)	Guards (Mean ± SD)	Typical Results per Game [6]
Number of Accelerations	98 ± 65	**178 ± 117**	**206 ± 76**	43–145
Number of Decelerations	**106 ± 67**	**183 ± 91**	**180 ± 72**	24–95
Number of High Accelerations	6 ± 5	**19 ± 20**	**23 ± 12**	1–15
Number of High Decelerations	7 ± 7	11 ± 8	18 ± 12	4–40

## Data Availability

The data presented in this study are available on request from the corresponding author. The data are not publicly available due to privacy concerns for the athletes included in this study.
